# Supplementary material for: The interactome of intact mitochondria by cross-linking mass spectrometry provides evidence for coexisting respiratory supercomplexes
Source: Mol Cell Proteomics. 2017 Dec 8;17(2):216–32. doi: 10.1074/mcp.RA117.000470 (PMC5795388; doi:10.1074/mcp.RA117.000470)
Supplement: Supplemental Data [file supp_RA117.000470_133922_0_supp_23978_fzffwf.zip › spectra_annotation/mito_DR_spectra_annotation/230-1-6-1-23-1.pdf]

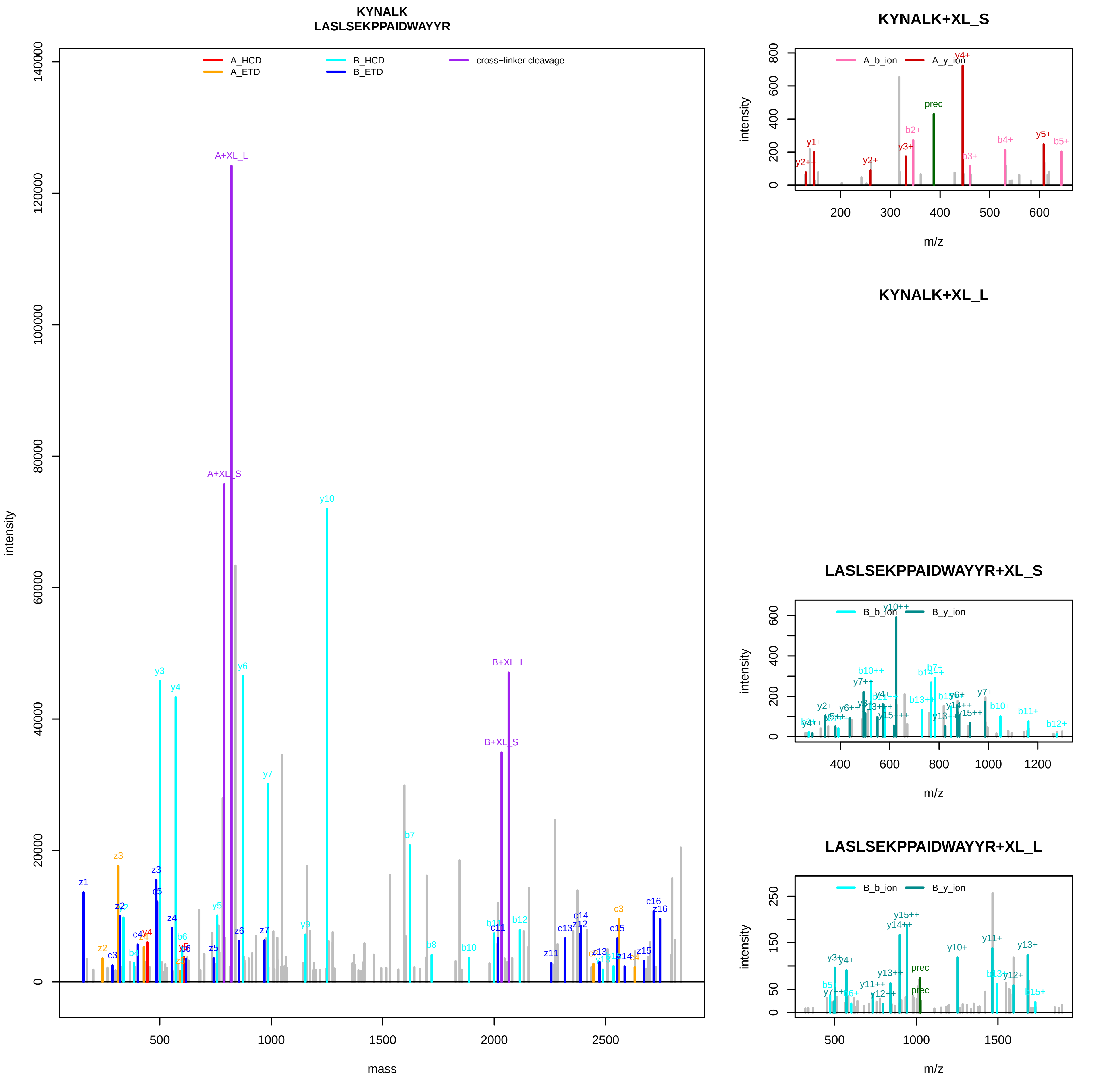
KYNALK
LASLSEKPPAIDWAYYR
A_HCD
A_ETD
B_HCD
B_ETD
cross−linker cleavage
A+XL_L
A+XL_S
B+XL_L
B+XL_S
y10
y3
y4
y6
y7
b7
z1
z3
c5
z2
c4
y4
z4
b6
z5
z6
z7
y5
y9
b8
b10
b11
c11
b12
z11
c13
c14
z12
z13
c15
c3
c16
z16
z15
z14
intensity
mass
KYNALK+XL_S
A_b_ion
A_y_ion
y4+
prec
b2+
y1+
y3+
y2+
y2++
b4+
y5+
b5+
b3+
intensity
m/z
KYNALK+XL_L
LASLSEKPPAIDWAYYR+XL_S
B_b_ion
B_y_ion
y10++
b10++
b7+
b14++
y7++
y7+
y2+
y4+
y6+
b10+
b11+
b12+
intensity
m/z
LASLSEKPPAIDWAYYR+XL_L
B_b_ion
B_y_ion
y15++
y14++
y10+
y11+
y13+
y3+
y4+
y13++
prec
y11++
y12++
y12+
b13+
b15+
y7++
b5+
b6+
intensity
m/z
